# Safety and immunogenicity of BK-SE36 in a blinded, randomized, controlled, age de-escalating phase Ib clinical trial in Burkinabe children

**DOI:** 10.3389/fimmu.2022.978591

**Published:** 2022-08-31

**Authors:** Edith Christiane Bougouma, Nirianne Marie Q. Palacpac, Alfred B. Tiono, Issa Nebie, Alphonse Ouédraogo, Sophie Houard, Masanori Yagi, Sam Aboubacar Coulibaly, Amidou Diarra, Takahiro Tougan, Amidou Z. Ouedraogo, Issiaka Soulama, Nobuko Arisue, Jean Baptiste Yaro, Flavia D’Alessio, Odile Leroy, Simon Cousens, Toshihiro Horii, Sodiomon B. Sirima

**Affiliations:** ^1^ Groupe de Recherche Action en Santé, Ouagadougou (GRAS), Ouagadougou, Burkina Faso; ^2^ Centre National de Recherche et de Formation sur le Paludisme (CNRFP), Ouagadougou, Burkina Faso; ^3^ Department of Malaria Vaccine Development, Research Institute for Microbial Diseases, Osaka University, Suita, Japan; ^4^ European Vaccine Initiative (EVI), Universitäts Klinikum Heidelberg, Heidelberg, Germany; ^5^ Department of Molecular Protozoology, Research Institute for Microbial Diseases, Osaka University, Suita, Japan; ^6^ Department of Infectious Disease Epidemiology, London School of Hygiene and Tropical Medicine (LSHTM), London, United Kingdom

**Keywords:** SE36, malaria blood-stage vaccine, serine repeat antigen, SERA5, *Plasmodium falciparum*, safety, immunogenicity

## Abstract

**Background:**

A blood-stage vaccine targeting the erythrocytic-stages of the malaria parasite *Plasmodium falciparum* could play a role to protect against clinical disease. Antibodies against the *P. falciparum* serine repeat antigen 5 (SE47 and SE36 domains) correlate well with the absence of clinical symptoms in sero-epidemiological studies. A previous phase Ib trial of the recombinant SE36 antigen formulated with aluminum hydroxyl gel (BK-SE36) was promising. This is the first time the vaccine candidate was evaluated in young children below 5 years using two vaccination routes.

**Methods:**

Safety and immunogenicity of BK-SE36 was assessed in a double-blind, randomized, controlled, age de-escalating phase Ib trial. Fifty-four Burkinabe children in each age cohort, 25–60 or 12–24 months, were randomized in a 1:1:1 ratio to receive three doses of BK-SE36 either by intramuscular (BK IM) or subcutaneous (BK SC) route on Day 0, Week 4, and 26; or the control vaccine, Synflorix^®^ *via* IM route on Day 0, Week 26 (and physiological saline on Week 4). Safety data and samples for immunogenicity analyses were collected at various time-points.

**Results:**

Of 108 subjects, 104 subjects (96.3%) (Cohort 1: 94.4%; Cohort 2: 98.1%) received all three scheduled vaccine doses. Local reactions, mostly mild or of moderate severity, occurred in 99 subjects (91.7%). The proportion of subjects that received three doses without experiencing Grade 3 adverse events was similar across BK-SE36 vaccines and control arms (Cohort 1: 100%, 89%, and 89%; and Cohort 2: 83%, 82%, and 83% for BK IM, BK SC, and control, respectively). BK-SE36 vaccine was immunogenic, inducing more than 2-fold change in antibody titers from pre-vaccination, with no difference between the two vaccination routes. Titers waned before the third dose but in both cohorts titers were boosted 6 months after the first vaccination. The younger cohort had 2-fold and 4-fold higher geometric mean titers compared to the 25- to 60-month-old cohort after 2 and 3 doses of BK-SE36, respectively.

**Conclusion:**

BK-SE36 was well tolerated and immunogenic using either intramuscular or subcutaneous routes, with higher immune response in the younger cohort.

**Clinical Trial Registration:**

https://pactr.samrc.ac.za/TrialDisplay.aspx?TrialID=934, identifier PACTR201411000934120.

## Introduction

Malaria is a huge public health problem. The significant decline in global morbidity and mortality rates achieved from 2000 to 2015 have largely stalled in recent years (1, 2). 95% of the estimated 241 million malaria cases in 2020 occurred in children living in sub-Saharan Africa (2). The Strategic Advisory Group on Malaria Eradication recommended the continuous development of improved vaccines to contribute to existing control strategies as well as future sustainable elimination (3).

Following the 6 October WHO recommendation for the first malaria vaccine, RTS,S/AS01, expanded use for children in moderate-to-high transmission settings, on 2 December 2021, Global Alliance for Vaccines and Immunizations (GAVI) approved the vaccine program for endemic countries across Africa (4, 5). RTS,S/AS01, the anti-sporozoite vaccine based on the *P. falciparum* circumsporozoite protein, is to be provided in a four-dose schedule to children from 5 months of age (5). In the multi-site phase III trial of this pre-erythrocytic vaccine, vaccine efficacy was 36.3% (95% CI 31.8–40.5) in 5–17 month old children who received 3 doses at 0, 1, and 2 months, plus a booster at 20 months (6). The vaccine does not confer sterile immunity and clinical malaria developed in the vaccinated population (6, 7). A vaccine that can control morbidity and possibly limit the next stages of human-to-mosquito transmission would be a valuable tool.

The *P. falciparum* serine repeat antigen 5 (SERA5) is an abundant essential blood-stage antigen secreted in large amounts into the lumen of the parasitophorous vacuole (8). Recent studies highlight its various roles from parasite egress to immune evasion. Conditional knockout of SERA5 caused a defect in the regulation of the lag phase that controls RBC membrane disruption, “explosive” rupture, and merozoite disposal (9). Interaction with calcium dependent protein kinase 1 (PfCDPK1) led to enhanced cytosolic Ca2+ levels that served as a trigger for merozoite egress (10). In addition, the N-terminal 47kDa fragment is bound to host vitronectin which in turn bound other host proteins camouflaging the merozoites against the host immune system (11).

A recombinant form of SERA5 N-terminal domain (SE36) was selected for clinical development based on: (a) epidemiological studies showing high antibody titers that inversely correlate with malaria symptoms and severe disease (12, 13); (b) *in vitro* studies demonstrating induction of antibodies that are inhibitors of parasite growth, exert antibody-dependent complement-mediated lysis of schizonts, or antibody-dependent monocyte-mediated parasite growth inhibition (12, 13); and (c) non-human primate challenge studies demonstrating protection against *P. falciparum* challenge infection (14). SE36 was prepared under Good Manufacturing Practice (GMP) standards, formulated with aluminum hydroxide gel (AHG) to yield BK-SE36. Phase I safety and immunogenicity trials of BK-SE36 were conducted in healthy, malaria naive Japanese adults (13), and in malaria exposed Ugandan volunteers aged 6- to 32-year-old (15). The encouraging results from the phase I trial in Uganda justified the conduct of a trial in younger cohorts, which we report here. The primary endpoints were the safety and reactogenicity of BK-SE36 administered subcutaneously or intramuscularly in healthy 12- to 60-month-old Burkinabe children. Secondary endpoints were humoral and cellular immune responses. This phase Ib trial provides safety and immunogenicity data with regards to two administration routes and the utility of a third dose at Week 26 (6 months after the first dose) for BK-SE36 in 1- to 5-year-old.

## Methods

### Trial site and population

The study was conducted at the Banfora trial centre of the Centre National de Recherche et de Formation sur le Paludisme (CNRFP). The Unité de Recherche Clinique de Banfora (URC-B), located about 400 km from Ouagadougou, Burkina Faso, is situated within the complex of the regional hospital. The trial participants were drawn from the Banfora Health Demographic Surveillance System (DSS) which covers a total population of 30,000 inhabitants. Bed net coverage in the area was around 80% (16) but indoor residual spraying (IRS) was considered inadequate or nil (17, 18). *P. falciparum* is responsible for 93% of malaria cases, with the rest attributed to monoinfections due to *P. malariae* (2%) and mixed infections of *P. falciparum* + *P. malariae* (5%) (19). The common vectors are *Anopheles gambiae*, *A. coluzzi* and *A. arabiensis* (16–18). Children under five years of age are the population subgroup of highest risk. Although transmission occurs throughout the year, about 60% of clinical cases are reported during June–September coinciding with the rainy months of May–November (19).

### Study design and objectives

This double-blind, randomized, controlled, age de-escalating, phase Ib clinical trial with a single-blind follow-up phase (Clinical trial registry PACTR201411000934120) enrolled 108 healthy, malaria-exposed African children. Children in both Cohort 1 (aged 25–60 months, n = 54) and Cohort 2 (aged 12–24 months, n = 54) were randomized into 3 treatment arms in a 1:1:1 ratio receiving: (a) 3 full doses of BK-SE36 by the subcutaneous route (BK SC), (b) 3 full doses of BK-SE36 by the intramuscular route (BK IM), and (c) 2 doses of the licensed *Pneumococcal* polysaccharide conjugate decavalent Synflorix^®^ vaccine, alternate with 1 dose of physiological saline by the IM route (control arm). The primary objective was to assess safety and reactogenicity; the secondary objective was to assess the immune response. For age de-escalation, Cohort 2 vaccination started after the recommendation of an independent safety monitoring committee (ISMC) who reviewed safety data from Cohort 1 up to and including 7 days of active follow-up post Dose 2.

The inclusion of a third dose at Week 26 (W26; Day 182) was intended to increase the immune response and evaluate the effect of a booster dose. The safety and immunogenicity of two doses was demonstrated in a previous trial in Uganda (15); while a three-dose regimen had been tested in malaria naïve Japanese adults (13). The common vaccination route in Japan, where the BK-SE36 vaccine was developed, is the subcutaneous route (SC). As the intramuscular route (IM) is the standard route of administration in the national Expanded Programme on Immunization, it was deemed important to add this treatment arm. Moreover, in some vaccines, IM administration is associated with a better immune response and fewer injection site reactions (20). The dosing interval of 28-days was similar to the minimum interval in vaccine doses according to the guidelines from the Advisory Committee on Immunization Practices (21). The sample size was calculated based on the safety objective. A group size of 18 subjects gives a minimum power of 85% to detect 1 or more SAEs that occur with a frequency of at least 10%. Allowing for losses to follow-up, a group size of 15 would still provide ≥79% power to detect 1 or more SAEs that occur with a frequency of at least 10%.

### Screening, enrolment, randomization, and blinding

Community consent was obtained from local village leaders and community members. Based on data from the DSS in the study area, all children aged 12- to 60-month-old and their parents/guardians were invited to local community meetings to explain the study. Those interested were invited to participate in a public lottery to randomly select participants for a screening visit. When the infant’s/child’s name was called, the parent/guardian randomly selected a sealed envelope containing “YES” or “NO”. “YES” would mean that the infant/child was invited for a screening visit. At the trial site, informed consent was sought and parent(s)/guardian(s) were asked to sign/thumbprint consent forms prior to performing any study related procedure. A literate, impartial witness was present for illiterate parent(s)/guardian(s).

Participants meeting the eligibility criteria (**Supplementary Material**) were assigned to treatment arms using a computer-generated randomization list. Randomization used permuted random block sizes of 6 and 9. For each cohort, allocation to a treatment number was based on the order that the subject presented for vaccination. The trial pharmacist opened sequentially numbered opaque sealed envelopes after ensuring that the child/infant before him met the eligibility criteria and had been given a study ID number. An independent vaccinator performed vaccine administration. All other study staff were blinded to treatment assignment. The trial remained double-blind until one month after the booster dose.

### Intervention, storage, and masking

BK-SE36 is produced by expressing recombinant SE36 based on amino acid residues 17–192 and 226–382 of *P. falciparum* (Honduras-1) SERA5 in *E. coli* BL21(DE3) pLysS (13). The purified protein was mixed with aluminum hydroxide gel in PBS. GMP-grade BK-SE36 vaccine was supplied by the Research Foundation for Microbial Diseases of Osaka University in single-dose vials as a lyophilized white powder that was reconstituted prior to vaccination (Lot number SER04B). When reconstituted with 1.3 mL of the supplied diluent (Japanese Pharmacopoeia water, Lot number D13T04), the opaque/opalescent liquid suspension contained 100 μg/mL of SE36 protein and 1 mg/mL aluminum. One mL was used for administration.

The control vaccine was Synflorix^®^ (GlaxoSmithKline Biologicals s.a.), a 10-valent adsorbed pneumococcal polysaccharide conjugate vaccine purchased locally in mono-dose, prefilled glass syringe (Lot numbers ASPNA361AA and ASPNA765AE). As per manufacturer recommendation, based on the participant age group, vaccinations (0.5 mL dose) were delivered with an interval of at least 2 months between the 2 primary doses: Synflorix^®^ was administered on Day 0 (D0) and Week 26 (W26); and physiological saline on Week 4 (W4). Physiological saline (Otsuka Pharmaceutical Factory, Inc) was supplied in twist-off type multi-dose plastic ampoules (Lot number K4J78).

All study vaccines were securely stored at 5 ± 3°C with limited access. To preserve blinding, masked, similar type syringes were used for vaccination (including for Synflorix^®^ and saline).

### Trial visits and safety assessments

Vaccinations were conducted on D0, W4/D28 and W26/D182. Following each vaccination, subjects were observed in the clinic for at least 60 minutes for any immediate local or systemic adverse events (AEs). Safety outcomes were also evaluated daily at home for the next 6 days, and during clinic visits on days 7 and 28 post vaccination. Daily contact visits were also done at D240–330 prior to clinic visit at D365. Monthly visits were conducted on D395–D455 by a field worker to check the participants’ status and refer them to the trial center, if necessary. The final clinic visit was at D477. During clinic visits, hematology safety tests included hemoglobin (Hb) and red cell indices (MCV, MCH, MCHC), white blood cell count (WBC) with differential absolute neutrophil count (ANC), RBC, and platelet count. Alanine aminotransferase (ALT), aspartate amino transferase (AST), total bilirubin, and creatinine were also assessed. The number and percentage of participants with AEs, serious adverse events (SAEs), AEs leading to withdrawal, and clinically significant hematological and biochemical abnormalities were recorded. The severity of AEs was assessed and evaluated based on a 3-grade scale (Grade 1 = mild, Grade 2 = moderate, or Grade 3 = severe) by the investigators (**Supplementary Material**). Fever, as presented here, was determined by measurement rather than reported history. A rapid diagnostic test and thick- and thin- blood smears were prepared whenever a subject presented with an axillary temperature of ≥ 37.5°C or a history of fever within the past 24 hours.

Malaria diagnosis was done by light microscopy in the Parasitology Unit at URC-B. Two thick and thin blood smears were prepared for each subject for samples obtained at D0, vaccination days (D28, D182), 28 days post vaccination (D56 and D210), D365, D477, and whenever clinical malaria was suspected at any unscheduled visit.

### Immunogenicity assessment

Anti-SE36 IgG antibody titers before vaccination (D0, D182), 4 weeks after each vaccination (D28, D56, D210), and at D365 and D477 were measured by ELISA. ELISA measurements, outsourced to a GLP certified testing facility (CMIC Pharma Science Co., Ltd., Japan), were performed using standardized methodology and expressed in titers calculated using an equilibrium line assay (13, 15). When clinical malaria was diagnosed during passive surveillance, samples for IgG analyses were obtained whenever possible for the initial unscheduled visit and one week after treatment. At each arm and each visit, the number and proportion of individuals with detectable SE36 IgG were reported. For serum samples with anti-SE36 IgG antibody levels below the limit of detection, a value of 8 was assigned for statistical analyses. Geometric mean titers were calculated at each time point. IgG1 and IgG3 subclasses were determined (22) for those with detectable anti-SE36 antibody titers 4 weeks after the second (W8/D56) and third (W30/D210) vaccinations.

T cell cytokine (IL-5, IL-13, and IFNγ) measurements for samples obtained before vaccination, 4 weeks post second (W8/D56) and third (W30/D210) vaccinations, and at D365 and D477 were done at the Immunology and Parasitology Laboratory at CNRFP using Human Th1/Th2 Cytokine ELISA Kit (Abcam, UK) according to manufacturer’s instructions. In brief, antibodies specific for IFNγ, IL-5 and 1L-13 were precoated respectively onto corresponding microtiter plates and samples, including standards of known concentrations, were incubated at room temperature for 2 h 30 min. The wells were then washed 3 times with PBS and antibody cocktails from the ELISA kits were added into wells, incubated at room temperature for 1h, washed and horseradish peroxidase conjugated streptavidin were added for 45 min. After removal of non-bound HRP conjugate, TMB substrate was added and incubated for 30 min in the dark at room temperature. Following incubation with stop solution, absorbance at 450 nm was measured with the microplate reader Biotek ELx808 (Winooski, Vermont 05404-0998 USA).

The mapping of protective epitopes in the SE36 antigen was done by ELISA (14) using serum samples obtained at W8/D56 and W30/D210 at the Department of Molecular Protozoology, Research Institute for Microbial Diseases (RIMD), Osaka University.

### Statistical methods

Statistical analyses were performed at the London School of Hygiene and Tropical Medicine using Stata ver 15 (Statacorp, College Station, TX, USA, www.stata.com). All safety analyses were descriptive in nature and presented as frequency distributions by vaccination group. For continuous variables, box-whisker plots, medians, inter-quartile ranges, and ranges were used. Antibody titers are presented in terms of the geometric mean for each time point and treatment arm. Two separate analyses were done: an analysis that included all subjects who received at least one vaccine dose and a separate analysis per protocol that included all subjects who received all three doses at the correct time interval. As similar results were obtained with both datasets, safety and immunogenicity data shown here are for all subjects who received at least 1 vaccination. Exact Binomial Proportion was used to estimate the proportion of subjects that received 3 doses without experiencing Grade 3 adverse events.

For comparison of anti-SE36 antibody titers, and fold-change in antibody titers in BK and SC arms, statistical tests (t-tests of log(titer)) were performed at 2 time points (D182, prior to Dose 3; D210, 4 weeks post Dose 3).

### Ethical and regulatory approval

The study was conducted according to the principles of the Declaration of Helsinki (2013), the ICH guidelines for GCP (CPMP/ICH/135/95) July 1996 (and its Revision 2 dated 9 November 2016), and in full conformity with relevant country regulations. Ethical reviews were conducted in Burkina Faso: Comité d’Éthique pour la Recherche en Santé du Burkina Faso (Ref: 2014-12-144) and Comité Institutionnel de Bioéthique du CNRFP (Ref: n°2014/071/MS/SG/CNRFP/CIB, N°2016/000008/MS/SG/CNRFP/CIB); Japan: Scientific Committee/Institutional Review Committee of the Research Institute for Microbial Diseases (Ref: 26-6), Osaka University (Ref: 574); and United Kingdom: London School of Hygiene and Tropical Medicine Research Ethics Committee (Ref: 9175). Approval for the clinical trial (N°2015:658/MS/CAB) and importation permit (N°20150016/MS/SG/DGPML/DRLP/SHPS/KKG) for the Investigational Products (IP) were obtained from Agence Nationale de Régulation Pharmaceutique (ARPN, previous name: Direction Générale de la Pharmacie, du Médicament et des Laboratoires (DGPML).

## Results

### Participant distribution, recruitment and demographic data

**Figure 1:**
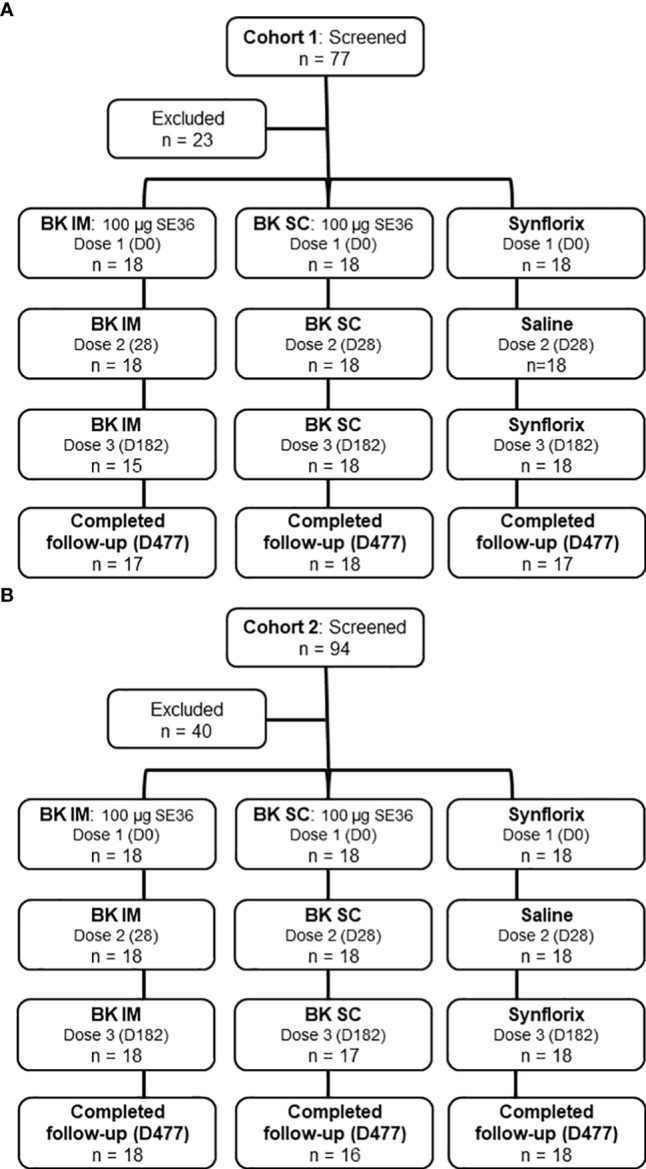
Trial profile. **(A)** Cohort 1 (25-60-month-old). The reasons for exclusion are: did not meet inclusion criteria (n=12), declined to participate (n=1) and sample size reached (n=10). **(B)** Cohort 2 (12-24-month-old). The reasons for exclusion are: did not meet inclusion criteria (n=33), declined to participate (n=2) and sample size reached (n=5). Results presented comes from all subjects who received at least 1 vaccination.

Seventy-seven children were screened for inclusion in Cohort 1 (25- to 60-month-old), of whom 54 were enrolled and randomized to three study arms each with 18 subjects (BK SC, BK IM, and control) (**Figure 1A**). Those who were excluded (n = 23) did not meet inclusion criteria (n = 12), declined to participate (n = 1), or were not enrolled because the sample size had already been achieved (n = 10). Seven days after Dose 2 of Cohort 1, the ISMC assessed the safety data prior to the Go decision to start the vaccination in Cohort 2 (12- to 24-month-old). For Cohort 2, of 94 subjects screened, 40 were excluded (n = 33, did not meet inclusion criteria; n = 2, declined to participate; n = 5, not enrolled because the sample size had already been achieved) (**Figure 1B**).

The vaccination of Cohort 1 started on July 4, 2015 (Dose 1) and the last vaccination (Dose 3) was completed by January 11, 2016. Follow-up was completed on Oct 28, 2016. Thirty-three children (92%) received all 3 doses of BK-SE36 and 18 (100%) received 2 doses of Synflorix (and physiological saline at Dose 2). Three subjects, all from the BK IM arm did not receive Dose 3 (n = 1, withdrew consent; n = 2, withdrawn by the investigator due to participation in another trial, and another due to erythema).

Vaccination of Cohort 2 began on October 12, 2015 (Dose 1) and was completed by April 18, 2016 (Dose 3). Follow-up was completed on February 7, 2017. Thirty-five children (97%) received all 3 doses of BK-SE36 and 18 (100%) received 2 doses of Synflorix (and physiological saline at Dose 2). One subject (BK SC) withdrew consent before Dose 3.

Demographic characteristics at enrolment among arms in each cohort were broadly similar (**Table 1**).

**Table 1:** Baseline characteristics of the study participants at enrollment within each vaccine arm.

Study cohorts		Cohort 1: 25-60 months				Cohort 2: 12 –24 months			
Arm		BK-SE36		Control (Synflorix)	Total	BK-SE36		Control (Synflorix)	Total
Route		SC	IM	IM	SC	IM	IM
n		18	18	18	54	18	18	18	54
**Gender**	Male	7 (39%)	7 (39%)	6 (33%)	20 (37%)	8 (44%)	6 (33%)	8 (44%)	22 (41%)
	Female	11 (61%)	11 (61%)	12 (67%)	34 (63%)	10 (56%)	12 (67%)	10 (56%)	32 (59%)
**Age** (months)	(mean ± SD)	43.7 ± 11.3	46.1 ± 9.6	47.8 ± 9.0	45.9 ± 10	18.5 ± 3.7	18.2 ± 3.1	19.3 ± 3.0	18.7 ± 3.3
**Heigh**t (cms)	(mean ± SD)	93 ± 7	96 ± 6	96 ± 7	95 ± 7	78 ± 4	77 ± 3	79 ± 4	78 ± 3
**Weight** (kgs)	(mean ± SD)	13.5 ± 1.8	14.8 ± 1.8	14.8 ± 2.2	14.4 ± 2.0	9.4 ± 1.0	9.0 ± 1.1	9.5 ± 1.4	9.3 ± 1.20
**Body mass index** (BMI)	(mean ± SD)	15.6 ± 1.1	16.0 ± 1.4	15.9 ± 1.4	15.8 ± 1.3	15.5 ± 1.6	15.0 ± 1.3	15.2 ± 1.2	15.2 ± 1.4

SC, subcutaneous route; IM, intramuscular route; n, no. of subjects.

### BK-SE36 safety and reactogenicity

In terms of immediate reactogenicity, within the first 60 minutes post-vaccination, abnormal pulse rates were reported in all vaccination arms of Cohort 1 and Cohort 2 after each vaccination (**Supplementary Table S1**). Grade 1 pain/limitation of limb movement was reported for one BK SC subject of Cohort 1 within 60 min of Dose 1. No other solicited local or systemic reactions were reported within the hour after each vaccination.

Local adverse events observed during the trial included pain at the injection site, swelling, erythema/redness, and induration (**Table 2**). Overall (Cohort 1 and 2 combined), the most commonly reported local events were induration (67%, 60%, and 53% for Dose 1, Dose 2, and Dose 3, respectively in BK-SE36 arms *vs* 53%, 17%, 22% for control arm) and pain (64%, 61%, and 26% for Dose 1, Dose 2, and Dose 3, respectively in BK-SE36 arms *vs* 67%, 22%, 14% in the control arm) (**Supplementary Table S2**). **Table 2** shows that, between arms, BK SC reported more AEs than BK IM (*e.g.* In Cohort 1: induration: 67%–89% for BK SC *vs* 0–44% for BK IM; pain: 67%–83% for BK SC *vs* 13–61% for BK IM; in Cohort 2: induration: 82%–100% for BK SC *vs* 17–56% for BK IM; pain: 12%–89% for BK SC *vs* 11%–44% for BK IM). Local events were either mild or moderate; and most resolved within 1–2 weeks without treatment. The longest induration resolved 44 days post Dose 1 for a BK SC subject in Cohort 2; the longest induration in the control arm resolved 27 days post Dose 1. Both were Grade 1 AEs. The longest recorded redness lasted for 158 days for 1 BK SC subject in Cohort 1 (Grade 1, observed after Dose 2) *vs* 2 days for control.

**Table 2:** Summary of local and systemic adverse events (full analysis set).

	Cohort 1 (25-60 months)								
	Dose 1			Dose 2			Dose 3		
	BK IM	BK SC	Control	BK IM	BK SC	Control	BK IM	BK SC	Control
n	18	18	18	18	18	18	15	18	18
**Local**									
Pain	9 (50%)*	13 (72%)	13 (72%)	11 (61%)	15 (83%)	6 (33%)	2 (13%)	12 (67%)	4 (22%)
Swelling	4 (22%)	2 (11%)	5 (28%)	6 (33%)	5 (28%)	1 (6%)	3 (20%)	1 (6%)	3 (17%)
Redness/Erythema	0	0	0	3 (17%)	5 (28%)	2 (11%)	0	5 (28%)	0
Induration	8 (44%)	12 (67%)	11 (61%)	7 (39%)	16 (89%)	2 (11%)	0	16 (89%)	2 (11%)
**Systemic**									
Fever	0	1 (6%)	2 (11%)	1 (6%)	1 (6%)	1 (6%)	0	0	0
Loss of appetite	0	0	0	1 (6%)	0	1 (6%)	0	0	0
Irritability/fussiness	0	0	0	0	0	0	0	0	0
Drowsiness	0	0	1 (6%)	1 (6%)	1 (6%)	1 (6%)	0	0	0
**Other AEs suspected to be related to study vaccine**									
Urticaria				1 (6%)					
	**Cohort 2 (12-24 months)**								
**n**	**18**	**18**	**18**	**18**	**18**	**18**	**18**	**17**	**18**
**Local**									
Pain	8 (44%)	16 (89%)	11 (61%)	8 (44%)	10 (56%)	2 (11%)	2 (11%)	2 (12%)	1 (6%)
Swelling	5 (28%)	4 (22%)	3 (17%)	6 (33%)	4 (22%)	0	3 (17%)	5 (29%)	4 (22%)
Redness/Erythema	0	7 (39%)	2 (11%)	0	4 (22%)	0	1 (6%)	3 (18%)	1 (6%)
Induration	10 (56%)	18 (100%)	8 (44%)	3 (17%)	17 (94%)	4 (22%)	6 (33%)	14 (82%)	6 (33%)
**Systemic**									
Fever	2 (11%)	0	2 (11%)	1 (6%)	2 (11%)	0	0	0	0
Loss of appetite	1 (6%)	1 (6%)	1 (6%)	1 (6%)	0	0	0	1 (6%)	0
Irritability/fussiness	0	0	0	0	0	0	0	1 (6%)	0
Drowsiness	0	0	1 (6%)	0	2 (11%)	0	0	0	0
**Other AEs suspected to be related to study vaccine**									
Pruritus	0	1 (6%)	0	0	0	0	0	0	0
Pyrexia	0	0	0	0	0	1 (6%)	0	0	0
Diarrhea	0	1 (6%)	0	0	1 (6%)	0	1 (6%)	0	1 (6%)
Vomiting	0	0	0	0	1 (6%)	0	0	0	0
Increased transaminase	0	0	0	0	1 (6%)	0	0	0	0

no. of children experiencing an event = n (% of children); BK IM = BK-SE36 via intramuscular route; BK SC = BK-SE36 via subcutaneous route

For systemic events, there were 3 Grade 3 fever events: from a control subject in Cohort 1 after Dose 1 (not related), a control subject in Cohort 2 after Dose 1 (possibly related) and a BK IM subject in Cohort 2 after Dose 2 (not related). Other AEs (loss of appetite, irritability and drowsiness) were less common (**Table 2**), generally mild and resolved within 3 days.

The most frequently reported AEs at any time during the trial period were respiratory tract infections (bronchitis, rhinitis, and cough) with Cohort 2 (younger cohort) having more events than Cohort 1. Gastrointestinal disorders (enteritis and gastroenteritis) were also common in Cohort 2 in all treatment arms (**Table 3**). Most of the AEs were due to common childhood illnesses. Only one related AE (**Table 2**), urticaria in a BK IM subject, was reported in Cohort 1. The AE occurred 2 days post Dose 2, was moderate in severity, and resulted in the discontinuation of the third dose (as per the investigator’s decision). The event resolved 5 days after onset. In Cohort 2, there were 8 mild to moderate AEs judged related to vaccination with no consequence on study continuation. Diarrhea was reported in four subjects (1 in BK IM, 2 in BK SC and 1 in control arm; one subject in addition to diarrhea reported vomiting), in all cases occurring within a day of vaccination and resolving ≤ 2 days after onset. Another BK SC subject had pruritus 4 days post Dose 1 which resolved 2 days after onset. One subject in the control arm had fever 2 days post Dose 2 which resolved (without treatment) 2 days after onset. High transaminasemia occurred 7 days post Dose 2 in one BK SC subject, of Grade 2 severity and resolved (without treatment) by 22 days after onset.

**Table 3:** Frequently reported adverse events.

	BK-SE36 IM		BK-SE36 SC		Control	
	Cohort 1	Cohort 2	Cohort 1	Cohort 2	Cohort 1	Cohort 2
	n = 18	n = 18	n = 18	n = 18	n = 18	n = 18
Bronchitis	11 (61%)* [19]	13 (72%) [39]	14 (78%) [26]	15 (83%) [41]	10 (56%) [20]	13 (72%) [29]
Rhinitis	11 (61%) [15]	13 (72%) [39]	10 (56%) [18]	16 (89%) [39]	11 (61%) [20]	14 (78%) [33]
Cough	4 (22%) [6]	0	3 (17%) [4]	3 (17%) [3]	3 (17%) [4]	0
Enteritis	0	9 (50%) [14]	0	7 (39%) [13]	2 (11%) [2]	7 (39%) [11]
Gastroenteritis	0	2 (11%) [2]	0	3 (17%) [3]	0	4 (22%) [4]

*no. of children experiencing an event (% of children), [total no. of events]

No serious adverse events (SAEs) were judged related to vaccination. Four (4) SAEs were reported in Cohort 1 (BK SC, n = 2 and control arm, n = 2) (**Supplementary Table S3**). Seven (7) SAEs were reported in Cohort 2 (BK IM, n = 3; BK SC, n = 2; and control, n = 2). All SAEs were due to severe malaria with most cases resolving within a week (longest around 10 days). Aside from SAEs, a Grade 3 event (high transaminasemia) occurred in Cohort 2 (BK SC) 160 days after Dose 3. The event was judged unrelated to vaccination and had resolved (without treatment) by 22 days after onset. The proportions of subjects that received three doses without experiencing Grade 3 adverse events were similar in all vaccination arms for both cohorts (Cohort 1: 100%, 89%, and 89% for BK IM, BK SC, and control, respectively; Cohort 2: 83%, 82%, and 83% for BK IM, BK SC, and control, respectively) (**Supplementary Table S3**). None of the SAEs or Grade 3 events resulted in the discontinuation of vaccination.

With regards to laboratory AEs, large variations, above or below the reference range, were observed in hematology and biochemistry parameters but most were considered to be clinically not significant and the child was well. In both cohorts, most out-of-range values in hematology were observed in platelets, MCV, MCH and ESR (additionally Cohort 2 has out-of-range values also in RBC and MCHC) but no strong evidence or pattern was repeated across treatment arms in both cohorts which could be interpreted as vaccine related. Some laboratory fluctuations led to or were correlated with AEs. In Cohort 1, fluctuations in Hb and platelet (n = 3) were linked to anemia (BK IM, n = 1; BK SC, n = 1; control, n = 2). Some elevated liver enzyme fluctuations were linked to high transaminasemia (BK SC, n = 2). Abnormal ALT, AST, and bilirubin values were also linked to hepatitis A (BK SC, n = 2; BK IM, n = 1). In Cohort 2, an abnormal Hb value was linked to anemia in the control group. Elevated liver enzymes were linked to high transaminasemia (BK SC, n=2; BK IM, n=1; control, n=1). In addition, elevated liver enzymes (ALT and AST) in 4 children in the control arm were also linked to hepatitis A.

### Humoral and cellular response to BK-SE36 vaccination

Geometric means titers (GMT) with 95% confidence intervals by vaccine arm and visit are shown in **Table 4**. Notably, detectable (> 8, the assigned value for statistical analyses), pre-vaccination anti-SE36 IgG antibodies were present in all arms at D0, prior to any vaccination (Cohort 1: 5/36 in BK arms (GMT for 5 subjects with detectable titers: 139.5, CI 50.2-387.6), 4/18 in the control arm (n = 4, GMT 216.4, CI 6.7-7012); Cohort 2: 17/36 in BK arms (n = 17, GMT 58.7, CI 37.4-92.2), 9/18 in the control arm (n = 9, GMT 52.8, CI 26.7-104.7)).

**Table 4:** Total anti-SE36 IgG antibody.

	Cohort 1					
	BK-SE36 Intramuscular		BK-SE36 Subcutaneous		Control (Synflorix^®^ + saline) Intramuscular	
	n	GMT (95% CI)	n	GMT (95% CI)	n	GMT (95% CI)
***Day 0***	18	9.4 (6.7, 13.0)	18	15.1 (8.0, 28.8)	18	16.6 (7.2, 38.5)
***Day 28***	18	18.2 (10.0, 33.1)	18	29.7 (15.7, 55.9)	18	29.7 (13.8, 63.9)
Day 56	18	97.2 (47.0, 200.9)	18	110.6 (63.6, 192.4)	18	28.5 (13.7, 59.3)
***Day 182***	17	33.1 (19.1, 57.3)	18	43.4 (23.5, 80.1)	18	28.5 (13.8, 58.9)
Day 210	17	155.3 (79.2, 304.6)	18	169.5 (92.6, 310.2)	18	24.0 (11.4, 50.5)
Day 365	17	27.9 (12.9, 60.4)	18	37.7 (20.5, 69.2)	17	18.0 (8.8, 37.0)
Day 477	17	38.3 (16.2, 90.7)	18	56.0 (27.5, 113.8)	17	43.4 (19.3, 98.0)
	**Cohort 2**					
***Day 0***	18	16.9 (9.9, 28.6)	18	24.9 (13.3, 46.9)	18	20.6 (11.6, 36.4)
***Day 28***	18	65.6 (38.0, 113.4)	18	63.7 (37.6, 107.8)	18	27.5 (15.2, 49.9)
Day 56	17	271.7 (144.5, 510.9)	16	304.0 (148.0, 624.6)	18	16.6 (10.4, 26.5)
***Day 182***	18	21.8 (10.7, 44.7)	17	29.4 (18.2, 47.6)	18	8.7 (7.6, 9.8)
Day 210	18	634.6 (284.3, 1416)	16	640.2 (374.8, 1093)	18	8.4 (7.6, 9.4)
Day 365	18	109.0 (50.3, 235.9)	16	93.9 (39.8, 221.2)	18	22.2 (13.4, 36.8)
Day 477	18	98.9 (39.7, 245.9)	16	38.6 (20.5, 72.9)	17	12.8 (8.9, 18.5)

Subjects were vaccinated at Day 0, 28 and 182; Day 28, 56 and 210 = 4 weeks after Dose1, 2, and 3, respectively; GMT = geometric mean titre (95% confidence interval); n = number of subjects;

Cohort 1: *p* = 0.50 for comparison of BK-SE36 arms at Day 182 (prior to Dose 3) and *p* = 0.83 for comparison of BK-SE36 arms at Day 210 (4 weeks post Dose 3).

Cohort 2: *p* = 0.48 for comparison of BK-SE36 arms at Day 182 (prior to Dose 3) and *p* = 0.99 for comparison of BK-SE36 arms at Day 210 (4 weeks post Dose 3).

In Cohort 1, 4 weeks after Dose 1 (D28) only a small rise in titers in both BK-SE36 arms was observed (**Table 4**). Four weeks after Dose 2 (D56), anti-SE36 IgG antibodies increased substantially in both BK-SE36 arms (10.4-fold change for BK IM, 7.3-fold change for BK SC) while the titers remained at a similar level in the control arm (1.7-fold change) (**Supplementary Table S4**). Titers waned at D182, before Dose 3 (6 months after Dose 1). Four weeks after Dose 3 (D210), the anti-SE36 IgG titers reached peak levels (16.5-fold change for BK IM, 11.2-fold change for BK SC). At D365 (26 weeks after Dose 3) and D477 (42 weeks after Dose 3), titers waned again to levels similar to D28 (28 days after Dose 1). There was no evidence of a difference when comparing GMTs between BK IM and BK SC using t-tests performed at 2-time points (before Dose 3 (D182): *p* = 0.50; 4 weeks post Dose 3 (D210): *p* = 0.83 for comparison between BK IM and BK SC) (**Table 4**). Antibody titers were relatively stable from D0 to D477 in the control group.

In the younger cohort, on D28, high anti-SE36 IgG antibody titers were seen in both BK-SE36 arms, indicating a good response immediately after Dose 1 (compared to Cohort 1) (**Table 4**). Fold-change after Dose 2 (D56) was comparable to the level of fold-change obtained from Cohort 1 after Dose 3 (D210) (**Supplementary Table S4**). Four weeks after Dose 3 (D210), the anti-SE36 IgG titers reached peak levels (37.6-fold for BK IM and 22.3-fold for BK SC). At D365 and D477 titers waned to levels slightly higher than D28 (28 days after Dose 1), except for the BK SC arm at D477 (**Table 4**). Again, there was no evidence of a difference when comparing GMT between vaccination routes using t-tests on log titers performed at 2-time points (before Dose 3 (D128): *p* = 0.48; 4 weeks after Dose 3 (D210): *p* = 0.99 for comparison between BK IM and BK SC). GMT and fold change in antibody titers in the control group remained relatively stable from D0–D477.

For serum samples with detectable anti-SE36 IgG antibody levels, IgG1 and IgG3 concentrations were measured 4 weeks after Doses 2 and 3 (**Table 5**). For both cohorts, it appears that BK-SE36 induced a more pronounced IgG1 subclass dominant response. Changes in IgG3 were not as marked as for the IgG1 subclass, although higher geometric mean concentrations were observed in Cohort 2.

**Table 5:** Concentration of anti-SE36 IgG1 and IgG3 subclasses.

		Cohort 1					
		BK-SE36 Intramuscular		BK-SE36 Subcutaneous		Control (Synflorix^®^ + saline) Intramuscular	
		n	GMC (95% CI)	n	GMC (95% CI)	n	GMC (95% CI)	
**IgG1**	Day 56	16	13.6 (7.2, 25.4)	17	14.0 (8.9, 22.0)	10	5.2 (1.5, 18.2)
	Day 210	16	16.1 (8.5, 30.4)	17	19.8 (11.9, 33.0)	8	4.7 (0.9, 23.3)

**IgG3**	Day 56	16	2.2 (0.5, 8.9)	17	1.0 (0.3, 3.7)	10	1.2 (0.2, 8.9)
	Day 210	16	1.9 (0.6, 6.7)	17	0.4 (0.1, 1.2)	8	4.7 (0.5, 46.9)
		**Cohort 2**					
**IgG1**	Day 56	17	28.7 (16.6, 49.9)	16	25.5 (12.7, 51.4)	8	4.0 (1.1, 15.1)
	Day 210	17	66.2 (37.9, 115.7)	16	57.5 (38.6, 85.6)	1	3.2

**IgG3**	Day 56	17	8.5 (2.9, 24.4)	16	9.7 (2.6, 36.9)	8	0.6 (0.1, 4.3)
	Day 210	17	3.5 (0.9, 13.1)	16	8.0 (2.9, 22.3)	1	0.1

Day 56, 4 weeks after Dose 2; Day 210, 4 weeks after Dose 3; GMC, geometric mean concentration (95% confidence interval); n, number of subjects.

Considering T-cell cytokines IL-5, IL-13, and IFNγ, several subjects in Cohort 1 did not show detectable levels at visits D56, D210, D365 and D477 (**Supplementary Table S5**). In Cohort 2, more subjects had detectable IL-5 and higher levels of IL-13, although these levels were highly variable and observed in all vaccine arms including the control.

Reactivity of serum to peptides covering the whole sequence of the SE36 protein showed that in both cohorts at D56 and D210, sera from all BK-SE36 vaccinees reacted most strongly with synthetic peptides 7, 8, 9, and 15 (**Supplementary Figure S1**). Control sera reacted most strongly to peptides 1, 7, 8, and 15.

## Discussion

The primary objective of the study was to assess the safety and reactogenicity of 3 full doses of BK-SE36 (100 µg SE36 protein with AHG as an adjuvant) when administered on D0, D28, and D182, by either subcutaneous or intramuscular route, in healthy African children naturally exposed to the parasite *P. falciparum*. The sample size was small, the immunological analysis descriptive in nature, and the study did not include functional assays or a comprehensive assessment of the cell-mediated immune response. However, this was the first study to assess safety and immunogenicity in an age group (12–60 months) that has not been included in previous vaccine trials of BK-SE36 and, likewise, to compare the immune response to vaccination using IM and SC routes for this vaccine candidate. Traditionally, aluminum adjuvanted vaccines are recommended for IM and live attenuated virus vaccines for the SC route (20). However, the common vaccination route in Japan, where BK-SE36 was developed, is SC. IM vaccinations are easier to perform, and this route remains the standard worldwide, with injections generally well tolerated. This trial shows that similar to Havrix^®^ (hepatitis A vaccine, inactivated) and Priorix-Tetra™ (measles–mumps–rubella–varicella [MMRV] combination vaccine), both routes are immunogenic (23, 24).

One hundred and four of 108 participants completed three vaccinations, and overall 68/72 (94%) children received all 3 vaccinations of BK-SE36. BK-SE36 showed an acceptable safety profile in this population of Burkinabe children. There were no serious adverse events, unexpected reactions or safety concerns considered to be related to BK-SE36 during the course of the trial. All SAEs (n = 11) reported were hospitalizations due to severe malaria judged not related to vaccination and most resolved in less than two weeks. The proportion of children that received three doses without experiencing Grade 3 adverse events were similar across vaccination arms in both cohorts.

Reactogenicity was similar to that seen in the Japanese phase Ia (13) and Ugandan phase Ib (15) clinical trials. The most frequently reported solicited local AEs were induration and pain, mostly mild to moderate in terms of severity. Although more (and longer) cases of local reactogenicity were reported in the BK SC arm than in the BK IM or the control arms, there was no apparent increase in AEs at Week 26 (Dose 3). No distinct differences were seen between the age groups. Other AEs related to vaccination (urticaria, pruritus, diarrhea, vomiting and high transaminasemia) were mild to moderate in severity. Overall, the safety profile was comparable to that of the control pneumococcal polysaccharide conjugate vaccine Synflorix^®^.

BK-SE36 induced a clear humoral immune response. Total anti-SE36 IgG antibody titers increased 4 weeks after Dose 2 and Dose 3 in the BK-SE36 arms in both cohorts. Overall, mean anti-SE36 protein antibody titer values were higher at these visits in the BK-SE36 arms compared to the control group and only the BK IM and BK SC arms showed >2.5-fold change in antibody titers after each vaccination. Cohort 2 (12–24 months) had 2-fold and 4-fold higher antibody titers than Cohort 1 after Dose 2 and Dose 3 of BK-SE36, respectively. The control arm, in both cohorts, had titers that remained low from Day 0–Day 577.

Some subjects (in Cohort 1, 5 children randomized in BK arms and 4 in control; Cohort 2, 17 children in BK arms and 9 in control) had anti-SE36 IgG antibodies pre-vaccination suggesting presence of residual maternal antibodies or exposure to *P. falciparum* infections early in life (25–27). In a surveillance study looking at antibody titers to merozoite antigens in children residing in both high (Banfora, Burkina Faso) and low (Keur Soce, Senegal) malaria transmission areas consistently low antibody titers were also observed (28). Although antibody titers were found to decline in the first few months, presumably due to the loss of maternal antibodies, the rate of waning of maternally derived and the degree of naturally acquired anti-SE36 IgG antibody titers (including seropositivity) has not been thoroughly studied in this age group; although age-related acquisition as a result of natural infection has been noted in previous studies (13, 27). Indeed, the first two doses for both age cohorts in this trial were administered during the rainy season (where malaria transmission is high), but high titers are not expected because of low immunogenicity and the clear age-dependency for IgG specific to SE36 (13). In the present study, in the absence of robust baseline data, it is not clear if existing pre-vaccination anti-SE36 IgG antibodies are due to natural exposure or are residual maternal antibodies.

SE36 was observed to tightly bind to host prote*in vitro*nectin that can act as a molecular camouflage (11) and thus repeated infections and presence of these vitronectin-bound SE36 complex could inevitably result to immune tolerance against SE36 molecule. In the Ugandan adult cohort, no significant increase in antibody titers were observed after 2 vaccinations of BK-SE36, in contrast to 6–10 year old Ugandan children where the proportion of subjects with >2-fold increase in antibody titers was 73% (15). In this trial, the proportion of subjects with >2-fold increase in antibody titers after Dose 2 was 83% for Cohort 1 and 79% for Cohort 2.

In BK-SE36 vaccinees, a booster vaccination (Dose 3) resulted in higher immune responses. This is in contrast to an earlier trial in Japanese adults that showed no significant difference in antibody titer post Dose 3 when compared to values obtained post Dose 2 (13). Differences in vaccination schedule may have contributed to the improved immunogenicity. In Japanese adults the three vaccinations were in 21-days interval, whereas, in both Burkinabe cohorts Dose 3 was delayed to Week 26 (182 days from Dose 1 or 154 days after Dose 2). Antibody levels dropped to near pre-vaccination titers 5 months after Dose 2, but 28 days post Dose 3, antibodies were boosted, sometimes to levels higher than those induced 28 days after two vaccinations. Following Dose 3, the proportion of subjects with >2-fold increase in antibody titers was 89% for Cohort 1 and 97% for Cohort 2. In trials of two other vaccine candidates, improved immunogenicity was also observed for a delayed dose schedule (29, 30).

After D210, GMT values drop at D365 (26 weeks after Dose 3; fold change in antibody titer compared to baseline was only 2.7-fold in Cohort 1 and 4.9-fold in Cohort 2) and D444 (42 weeks after Dose 3; Cohort 1 = 3.9-fold change, Cohort 2 = 3.1-fold change); but not to levels similar to the control arm (Cohort 1, D365 = 1.0-fold change, D444 = 2.5-fold change; Cohort 2, D365 = 1.1-fold change, D444 = 0.6-fold change). Notably, the high transmission season was 6 months after Dose 3 for Cohort 1 and 2 months after Dose 3 for Cohort 2. Improvement in vaccination schedules (timing of third dose) should also be explored. It is envisioned that protection can be obtained when high anti-SE36 antibody titers induced by vaccination (4 weeks after Dose 3) coincide with the time of greater risk of contracting malaria (*i.e*., during the rainy season when transmission is highest).

No marked difference was observed when BK-SE36 was administered *via* the intramuscular or subcutaneous route of the vaccination. As previously reported, the vaccine induced response was composed mostly of IgG1 (22). With regards to epitope mapping, binding was observed in all arms to peptide 15, the binding site of the host protein, vitronectin. This binding property has been found to be more or less conserved in global *P. falciparum* isolates (11). Control sera reacted also to peptide 1. Synthetic peptide 1 lies in the intrinsically unstructured octamer repeat region at the N-terminal domain of SE36. All BK-SE36 arms reacted most strongly with peptides 7, 8, and 9 which corresponds to domains in the middle of the SE36 molecule proximal to the serine repeat region (14). Binding to peptide regions that lie in characteristically disordered or intrinsically unstructured regions (*e.g*. peptides 1, 7, 8, 9) further implies the absence of a strict conformational requirement for SE36 to be able to elicit an immune response (14).

The contribution of SE36 antigen-specific helper T cells remains unclear as cytokine secretion levels were low overall for IL-5, IL-13 (used as Th2 response markers) and IFNγ (as a Th1 response marker). The proportion of subjects with cytokine responses was higher in the younger cohort (Cohort 2), but no marked associations were found in relation to vaccine arm. Aside from small sample size, the wide variability seen may be attributed to an immature immune system, short-lived responses or very low response levels that fall below the threshold of detection in peripheral blood sampling. Only three T cell response markers were used and further studies are needed to cover other cytokines in the cellular immune response repertoire. Studies investigating protective efficacy against malaria infection and clinical disease are needed for more robust conclusions. So far, previous trials (13, 15, 22) and sero-epidemiological studies (12–14) suggest some subtle differences in antibody IgG subclass profile, fine epitope specificity, and potential differences in T-helper cell responses between immune response observed in vaccinees and immune response observed as a result of natural infection. These differences need to be further explored. Nevertheless, in congruence with prior trials, BK-SE36 is a promising blood-stage malaria vaccine candidate.

## Conclusions

BK-SE36 malaria vaccine appears to be well-tolerated when given to healthy semi-immune 12-60 month old children in Burkina Faso, at the dose of 100 µg, subcutaneously or intramuscularly on Days 0, 28, and 182. Although, BK-SE36 was immunogenic in both cohorts, whichever administration route was used, the IM route appears to have a lower risk of adverse reactions at the site of vaccination than the SC route. Moreover, younger children (12–24 months old) showed better immune response. The third dose at Week 26 boosted the humoral response to BK-SE36 in both age cohorts. This study supports the design and conduct of a phase IIb double-blind study in children under 5 years.

## Data availability statement

The original contributions presented in the study are included in the article/**Supplementary Material**. Further inquiries can be directed to the corresponding authors.

## Ethics statement

The studies involving human participants were reviewed and approved by Comité d’Éthique pour la Recherche en Santé du Burkina Faso (Ref: 2014-12-144) and Comité Institutionnel de Bioéthique du CNRFP (Ref: n°2014/071/MS/SG/CNRFP/CIB, N°2016/000008/MS/SG/CNRFP/CIB) (Burkina Faso); Scientific Committee/Institutional Review Committee of the Research Institute for Microbial Diseases (Ref: 26-6), Osaka University (Ref: 574) (Japan); and London School of Hygiene and Tropical Medicine Research Ethics Committee (Ref: 9175) (United Kingdom). Approval for the clinical trial (N°2015:658/MS/CAB) and importation permit (N°20150016/MS/SG/DGPML/DRLP/SHPS/KKG) for the Investigational Products (IP) were obtained from Agence Nationale de Régulation Pharmaceutique (ARPN, previous name: Direction Générale de la Pharmacie, du Médicament et des Laboratoires (DGPML). Written informed consent to participate in this study was provided by the participants’ legal guardian/next of kin.

## Author contributions

SS, EB, AT, IN, OL, SH, FD, NP, and TH contributed to the conception and design of the study. EB, AT, IN, AO, SaC, AD, IS, JY, and SS were responsible for study implementation at the study site. AZO organized the database. SiC performed the statistical analysis. TH, NP, SH, and SS analyzed the data. MY, NA, TT, and NP for materials/reagents/assay development and analysis tools. EB and NP wrote the first draft of the manuscript. All authors contributed to manuscript revision, read, and approved the submitted version.

## Funding

This work was supported by the Global Health Innovative Technology Fund (G2014-109) awarded to the Consortium; MEXT KAKENHI Grant Numbers 15651988, 24249024, Funds for Integrated Promotion of Social System Reform and Research and Development (38201103-01), Grant for Translational Research Network Program (JP20lm0203135, AMED) (C-13) awarded to TH; Grant for Cyclic Innovation for Clinical Empowerment by AMED (17nk0101206j0003) to Nobelpharma Co., Ltd (NPC, Tokyo, Japan); and Irish Aid to EVI. An additional in-kind contribution was also obtained from NPC. The funders were not involved in the study design, collection, analysis, interpretation of data, the writing of this article or the decision to submit it for publication.

## Acknowledgments

We thank study volunteers and their families for participation in the study and acknowledge the support of the URCB Centre (Unite de Recherche Clinique de Banfora), the Local Safety Monitor and Independent Safety Monitoring Committee for their support (Ken J. Ishii, Yoshio Hirota, Meta Roestenberg, Kwaku Poku Asante and Marceline T. Yaméogo). Guidance of the regulatory authorities and ethics committees in Burkina Faso, UK, Japan; and the Independent Scientific Advisory Committee (Carole Long, Mahamadou Thera, Takafumi Tsuboi and Chetan Chitnis) during the trial and follow up study are much appreciated. We also gratefully acknowledge the clinical trial assistance of Lydia Dabré (GRAS), the quality assurance assistance of Nathalie Imbault (EVI), laboratory assistance of Hideko Yoshikawa, Sawako Itagaki and Yuko Oishi (RIMD); the administration and logistical support of Nadège Kinda (GRAS), Sten Larsen Finnsson and Thorsten Kohaut (EVI), and Kumiko Tai (RIMD). Vaccine manufacture and supply was supported and undertaken by The Research Foundation for Microbial Diseases of Osaka University (BIKEN), Japan. The trial was sponsored by NPC; logistics, support and all other activities are duly acknowledged from Masanori Osakabe, Takako Aburada, Taka Sato, Satoru Murayama, and Motomichi Kouno.

## Conflict of interest 

TH is the inventor of BK-SE36 and all rights have now been turned over to NPC. NP served as contract researcher for NPC, Apr - Sept 2017. SH, FD and OL received support from NPC for salaries, travel and CRO cost for clinical monitoring. EB, SaC, AD, AZO, JY also received support from NPC for salaries during the long term follow-up. 

The remaining authors declare that the research was conducted in the absence of any commercial or financial relationships that could be construed as a potential conflict of interest.

## Publisher’s note

All claims expressed in this article are solely those of the authors and do not necessarily represent those of their affiliated organizations, or those of the publisher, the editors and the reviewers. Any product that may be evaluated in this article, or claim that may be made by its manufacturer, is not guaranteed or endorsed by the publisher.
