# Supplementary material for: Safety and immunogenicity of BK-SE36 in a blinded, randomized, controlled, age de-escalating phase Ib clinical trial in Burkinabe children
Source: Front Immunol. 2022 Aug 31;13:978591. doi: 10.3389/fimmu.2022.978591 (PMC9471861; doi:10.3389/fimmu.2022.978591)
Supplement: Supplementary file 1 [file DataSheet_1.pdf]

Supplementary Material

1 Protocol Synopsis

**Title**	Safety and immunogenicity evaluation of recombinant *E. coli* BK-SE36 malaria vaccine candidate with aluminium hydroxide gel as adjuvant administered either subcutaneously or intramuscularly in healthy malaria exposed African children living in Burkina Faso: A double blinded, randomized, controlled, age de-escalating, phase Ib clinical trial followed by an extended single blind follow up phase.
**Trial Identifier**	BK-SE36/003
**Principal Investigator**	Dr. Sodiomon Bienvenu Sirima, BA, MD, PhD<br>Centre National de Recherche et de Formation sur le Paludisme (CNRFP)
**Active ingredient**	When reconstituted with 1.3 mL of the supplied diluent, the solution contains 100 µg of *P. falciparum* SE36 protein
**Study population**	Healthy malaria exposed African children living in Burkina Faso
**Trial Centre**	Centre National de Recherche et de Formation sur le Paludisme (CNRFP)<br>Unité de Recherche clinique de Banfora (CNRFP/URC-B) 01 BP 2208<br>Ouagadougou 01 Burkina Faso
**Planned Trial Period**	Q2-2015 to Q1-2017. Total duration: 22 months
**Enrolment period**	8 weeks for each cohort.
**Rationale**	**Background information on the disease to be treated**<br>Malaria causes a huge global public health problem with around 40% of the world's population at risk of infection. There are approximatively 200 million clinical episodes of *Plasmodium falciparum* malaria and more than 600 000 people die from the disease annually. In Africa, a child dies from malaria every minute.<br>A malaria vaccine is crucial in the face of continued high malaria transmission, increasing drug and insecticide resistance, and inadequate coverage of current control interventions. Currently, no effective vaccine against malaria exists.<br><br>**Background information on the BK-SE36 vaccine**<br>There is a strong justification for blood-stage vaccines since protection by the anti-sporozoite RTS,S/AS01 vaccine candidate, currently the most advanced malaria vaccine, is not complete and long lasting. Asexual-stage parasites cause symptomatic malaria and blood-stage antigens are targets of acquired immunity. Controlling parasite density may reduce disease severity and *P. falciparum* gametocyte density, hence, potentially reducing disease transmission.<br>The *Plasmodium falciparum* serine repeat antigen-5 (SERA5) is an abundant blood-stage antigen secreted in large amounts into the lumen of the parasitophorous vacuole. It plays an essential role in the parasite life cycle and was among the first

	physiological substrate identified for a serine protease implicated for parasite egress.<br>A recombinant form of SERA5 N-terminal domain (SE36) was selected for clinical development on the basis of the following: (i)epidemiological studies showing high antibody titres that inversely correlate with malaria symptoms and severe disease; (ii) *in vitro* studies demonstrating induction of antibodies that are inhibitors of parasite growth, exert antibody-dependent complement-mediated lysis of schizonts, or antibody-dependent monocyte-mediated parasite growth inhibition; and (iii) animal studies demonstrating protection against *P. falciparum* challenge in non-human primates.<br>SE36, was prepared under current Good Manufacturing Practice (cGMP) constraints and formulated with aluminium hydroxide gel to yield BK-SE36.<br>The safety and immunogenicity of BK-SE36 was demonstrated in a phase Ia trial in malaria naive Japanese adults; and in a phase Ib trial conducted in healthy subjects aged 6–32 years from a malaria endemic area in Northern Uganda. The trial promising results justifies the conduct of a trial of BK-SE36 in younger cohorts in endemic areas.<br><br>**Trial rationale**<br>A blood-stage vaccine is also desirable to deal with epidemic transmission patterns as it is uncertain how changing transmission patterns may impact malaria disease severity. Conducting a Phase Ib trial in Burkina Faso will allow for (i) testing of the vaccine candidate in a younger age group (1-5 years old), (ii) generation of additional information/data on safety, immunogenicity and preliminary possible efficacy, and (iii) comparison of clinical trial results from two different African countries with different malaria endemicity -Uganda and Burkina Faso-.
**Objectives**	**Primary**<br>Assess the safety and reactogenicity of 3 full doses of BK-SE36 (100 μg SE36 protein) malaria vaccine candidate with aluminium hydroxide gel (AHG) as adjuvant, in either subcutaneous or intramuscular route, in healthy African children exposed to the parasite *Plasmodium falciparum*. The adverse event grading for the clinical abnormalities will be done according to the Brighton collaboration guidelines (www.brightoncollaboration.org).
	**Secondary**<br>• Assess the humoral immune response to the vaccine antigens administered subcutaneous or intramuscularly by measuring the level of IgG<br>• Assess the quality of the humoral immune response by measuring IgG1, IgG3 subclasses<br>• Assess T cell cytokines IL-5, IL-13, and IFNγ production.
	**Exploratory**<br>• Assess the efficacy of the vaccine by measuring incidence of clinical malaria among the groups of children from 4 weeks after the second vaccine dose until the last visit. A finger prick blood sample will be collected for preparation of

thick and thin blood smears for microscopic examination in the event of fever (tympanic temperature ≥38.0°C) or history of fever in the past 24 hours.

- Assess the efficacy of the vaccine against asymptomatic carriage of *P. falciparum*. Blood smears will be collected to assess the proportion of individuals carrying asexual *P. falciparum* malaria parasites by microscopy at different time points.
- Assess the efficacy of the vaccine against gametocytes carriage. RNA samples will be collected to assess the proportion of individuals carrying gametocytes using molecular methods
- Assess the effects of natural boosting on immune responses to the vaccine occurring after the second immunisation by measuring the IgG levels one week after clinical malaria diagnosis
- Assess the quality of the humoral immune response on all subjects by mapping the protective epitope(s) in SERA5.

Trial design

Double blinded, randomized, controlled, age deescalating, phase Ib clinical trial followed by an extended single blind follow up phase.
One hundred and eight (108) healthy subjects will be included into two cohorts of malaria exposed African children. Cohort 1 will consists of children aged 25-60 months (n=54) and cohort 2, children aged 12-24 months (n=54).
Subjects of each cohort will be randomised into three arms in a 1:1:1 ratio.

Cohort	*Arm*	*Administration route*	*Number of subjects*
1: Children aged 25-60 months	1. BK-SE36	Subcutaneous	18
	2.BK-SE36	Intramuscular	18
	3.Control vaccine	Intramuscular	18
2: Children aged 12 - 24 months	1.BK-SE36	Subcutaneous	18
	2.BK-SE36	Intramuscular	18
	3.Control vaccine	Intramuscular	18
Total			108

Rationale for the number of doses: 2 vaccinations (28 days apart) + 1 booster dose: Immunogenicity results from Japanese vaccinees suggest that 2 administrations were enough for 100% seroconversion. However, results of the phase Ib trial in Uganda only showed 72% seroconversion in 6-10 years-old. Inclusion of a booster dose at week 26 is intended to increase immunogenicity response of the malaria exposed population. This trial would also allow evaluation of the effect/role of a booster dose.
Safety and immunogenicity of doses 21-days apart was confirmed in phase Ia and phase Ib trial. A dosing interval of 28-days would be similar to schedules of inactivated vaccines.

	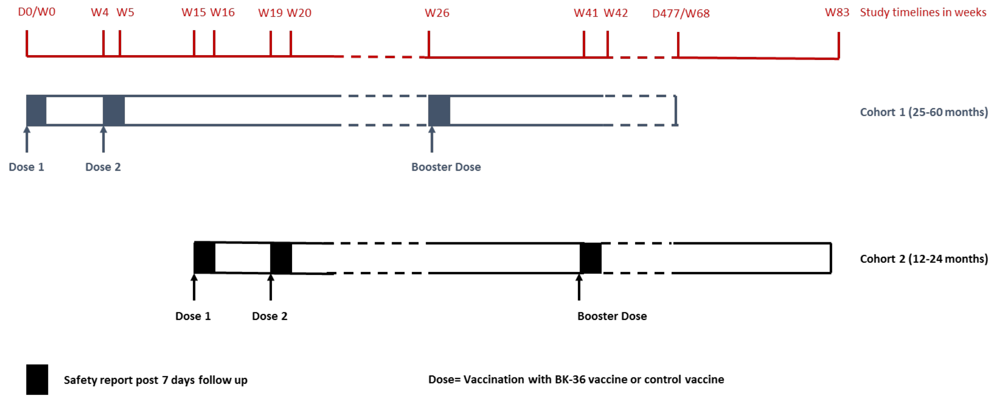
**GO/No GO**	The age de-escalation GO/No GO criteria will be based on safety criteria:<br>No GO: Stopping rule will be: any Serious Adverse Event (SAE) related to vaccination or 50% of subjects had Grade 3 adverse reaction persisting at Grade 3 for > 48 hours during the 7 follow-up days.<br>The threshold of 50% corresponds to a consensus decision in accordance with the African Malaria Network Trust (AMANET) Malaria vaccine guidelines and will be applied for the start of the vaccination of cohort 2. Nevertheless, the final decision for GO/No GO will belong to the sponsor or sponsor representative according to the recommendations of an Independent Safety Monitoring Committee (ISMC). The recommendations of the ISMC will be based on a safety report that will be prepared after 7 days of active follow-up after the second vaccination of cohort 1.<br>If the safety profile of any arm of a cohort meets the No GO criteria described above, then all the immunisations of the volunteers in this arm will be stopped.<br>GO/No GO criteria for immunogenicity: Malaria exposed subjects have different levels of pre-existing immunity resulting from natural exposure. There is no immunogenicity stopping rule to proceed from cohort 1 to cohort 2 and it is expected to see higher immune response in children 12-24 month-old.
**Inclusion Criteria**	**Specific inclusion criteria for cohort 1 (25-60 months)**<br>• Female or male subject aged 25-60 months inclusive at the time of the first vaccination<br>**Specific inclusion criteria for cohort 2 (12-24 months)**<br>• Female or male subject aged 12-24 months inclusive at the time of first vaccination<br>**Common inclusion criteria for cohort 1 and 2**<br>• Residing within the Banfora health district and planning to stay for the study duration.<br>• Appear to be in generally good health based on malnutrition index and clinical and laboratory investigation<br>• Signed or thumb-printed informed consent obtained from the parent(s)/guardian(s) of the child. Where parent(s)/guardian(s) are illiterate, the consent form will be countersigned by an impartial witness.<br>• Subjects who the investigator believes that their parents/guardians can and will comply with the requirements of the protocol (e.g. return for follow-

	up visits) should be enrolled in the study. The trial period for each subject is ca 18 months.
**Non-inclusion criteria**	**For cohort 1 and 2**<br>• Previous participation in any malaria vaccine trial<br>• History of blood transfusion within the last 3 months<br>• Symptoms, physical signs or laboratory values suggestive of systemic disorders, including renal, hepatic, cardiovascular, pulmonary, skin, immunodeficiency, psychiatric and other conditions, which could interfere with the interpretation of the trial results or compromise the health of the volunteers<br>• Any clinically significant laboratory abnormalities on screened blood samples outside the normal range, as defined at the clinical trial site. Specifically:<br>o Haemoglobin less than 8.0 g/dL,<br>o Serum Creatinine concentration greater than 70 μmol/L,<br>o Serum ALT concentration greater than 45 U/L,<br>o Low platelets count (< 100,000/mm^3)<br>• Immunosuppressive therapy (steroids, immune modulators or immune suppressors) within 3 months prior to recruitment. (For corticosteroids, this will mean prednisone, or equivalent, ≥ 0.5 mg/kg/day. Inhaled and topical steroids are allowed.)<br>• Any confirmed or suspected immunosuppressive or immunodeficiency condition based on medical history and physical examination (No testing will be done for HIV)<br>• A family history of congenital or hereditary immunodeficiency<br>• Major congenital defects<br>• Subjects with splenectomy<br>• History of anaphylaxis or known severe hypersensitivity to any of the vaccine components (adjuvant or antigen or excipient)<br>• Administration of gamma globulin: 4 weeks prior and after each vaccination if administration is necessary during the study period, the volunteer will be withdrawn from the study<br>• Planned administration/administration of a vaccine not foreseen by the study protocol within 30 days of the first dose of vaccine(s)<br>• Use of any investigational or non-registered drug or vaccine within 30 days preceding the first dose of study vaccine, or planned use during the study period<br>• Weight-for-age Z score of less than –3 or other clinical signs of malnutrition

	• Current participation in another clinical trial, or within 12 weeks of this study<br>• Any other finding which in the opinion of the investigators would increase the risk of an adverse outcome from participation in the trial or result in incomplete or poor quality data.
**Holding and stopping rules**	The trial may be placed on safety hold for the following reasons:<br>• On advice of the safety monitor.<br>• On advice of the investigator.<br>• On advice of the ethics committee or the ISMC.<br>• One or more participants experience a serious adverse event (SAE) that is determined to be related to BK-SE36 vaccine administration.<br>• In case of SAE possibly related to BK-SE36 vaccination, the immunisation of the remaining subjects will be immediately (but not finally) discontinued until the decision of the sponsor or the sponsor representative according to the ISMC recommendation. The ISMC will be held within 48 h following the SAE to conclude if the causality of the event was unrelated or related to the vaccine. The ISMC will recommend stopping, pausing and continuing the immunisation. The vaccination will only be resumed upon the decision of the sponsor representative according to the ISMC's recommendations.<br><br>The trial will be stopped for the following reason:<br>• One or more participants experience a Suspected Unexpected Serious Adverse Reaction (SUSAR) that is related to study vaccines administration.
**Investigational products**	BK-SE36 is recombinant SE36 protein expressed in *E. coli*, adsorbed to aluminium hydroxide gel (AHG)
	**Form**<br>Lyophilized; reconstitution with supplied diluent prior to administration
	**Dosage**<br>100 μg/mL *P. falciparum* SE36 protein, 1 mL
	**Adjuvant**<br>Aluminium 1,000 μg/mL
	**Route of administration**<br>Subcutaneous or intramuscular
**Control product**	Pneumococcal polysaccharide conjugate vaccine Synflorix® in alternance with physiological saline solution (NaCl 0.9%)

Form
Synflorix® : suspension for injection
Saline solution: liquid

Dosage
1 dose (0.5 mL) contains:

Pneumococcal polysaccharide serotype	Quantity
1[1,2]	1 µg
4[1,2]	3 µg
5[1,2]	1 µg
6B[1,2]	1 µg
7F[1,2]	1 µg
9V[1,2]	1 µg
14[1,2]	1 µg
18C[1,3]	3 µg
19F[1,4]	3 µg
23F[1,2]	1 µg

[1] adsorbed on aluminium phosphate 0.5 mg Al^{3+}
[2] conjugated to protein D (derived from non-typeable *Haemophilus influenzae*) carrier protein 9-16 µg
[3] conjugated to tetanus toxoid carrier protein 5-10 µg
[4] conjugated to diphtheria toxoid carrier protein 3-6 µg

Route of administration
Intramuscular

Vaccination Schedule

	Study Cohorts			
	1.Children aged 25-60 months		2.Children aged 12 – 24 months	
	**Vaccine arm**	**Control arm**	**Vaccine arm**	**Control arm**
**Schedule**	N=36	N=18	N=36	N=18
**Day 0**	BK-SE36	Synflorix®	BK-SE36	Synflorix®
**Week 4**	BK-SE36	Physiological saline	BK-SE36	Physiological saline
**Week 26**	BK-SE36	Synflorix®	BK-SE36	Synflorix®

Number of scheduled Visits
11 visits on site (+25 contact visits).

Follow-up duration
Total duration: 16 months following the first vaccination

Serology Schedule
At screening, Day 0 (before 1st administration), Week 1, 4 (before 2nd administration),5, 8, 26 (before booster dose), 27, 30, 52 and 68.

**Sample size justification**	The sample size is calculated to address the primary objective (safety). With 18 children in each arm, the study has a minimum power of 85% for detecting one or more SAE that occur with a frequency of at least 10%.
**Endpoints**	**Primary**<br>Number of adverse events (Grade 1–3 and serious adverse events) possibly, likely and definitely related to vaccination
	**Secondary**<br>• Anti-SE36 protein antibody titre at Day 0, Week 4, 8, 26, 30, 52 and 68.<br>• Concentration of SE36 specific IgG1, IgG3 concentration one month after the second vaccination (Week 8); and one month after the booster dose (Week 30)<br>• Concentration of T cell cytokines IL-5, IL-13, and IFNγ by ELISA at Day 0, Week 8, 30, 52 and 68.
	**Exploratory**<br>• Incidence of clinical malaria from 4 weeks after the second immunisation until the last visit based on different cases definitions:<br>-Primary case definition: Documented fever (tympanic temperature ≥ 38.0°C with asexual parasites density ≥5,000/μL)<br>-Secondary cases definition: Fever/history of fever with asexual parasites density at different cut-offs: 500/μL, 2,500/μL, 20,000/μL)<br>• Proportion of individuals carrying asexual parasites by microscopy at any density at Day 0 (before first administration), Week 4 (before second administration), 8, 26 (before booster dose), 30, 52 and 68<br>• Proportion of individuals carrying gametocytes by PCR at Day 0 (before first administration), Week 4 (before second administration), 8, 26 (before booster dose), 30, 52 and 68<br>• Anti-SE36 protein antibody titre one week after a malaria episode from the second vaccination until the last visit<br>• Determining protective epitopes of SERA5 one month after the second vaccination (Week 8) and booster dose (Week 30)
**Primary evaluation criteria**	The safety profile will be assessed by the following criteria:<br>• Immediate reactogenicity (reactions within 60 minutes after each vaccination)<br>• Local and systemic reactogenicity measured from Day 0 to Day 7 after each vaccination<br>• Any unsolicited adverse event within one month after each vaccination<br>• Any Serious Adverse Event (SAE) occurring throughout the study duration starting from the first immunisation.<br>The relationship of the adverse event to the vaccine will be established by the investigator as definitely, probably, possibly, unlikely related, or not related.

	• Occurrence of clinically significant hematological and/or biochemical abnormalities by laboratory test, one week and four weeks after each vaccination, in reference with the baseline before the first dose, by measuring:<br>o RBC, hemoglobin, MCV, MCH, MCHC, platelets, ESR and WBC with differential counts.<br>o AST, ALT, total bilirubin, creatinine
**Secondary evaluation criteria**	• The humoral immune response to vaccination will be assessed by measuring the titre of SE36 specific IgG by ELISA on samples obtained before first vaccination at Day 0 and Week 4, 8, 26, 30, 52 and 68<br>• IgG1 and IgG3 subclasses by ELISA on samples obtained at Week 8 and Week 30<br>• The T cell cytokines IL-5, IL-13, and IFNγ production by ELISA on serum samples at Day 0, Week 8, 30, 52 and 68.
**Exploratory evaluation criteria**	• Incidence of clinical malaria assessed by microscopic examination of thick and thin blood smears in the event of fever (Tympanic temperature ≥ 38.0°C) or history of fever in the past 24hours, starting from 4 weeks after the second immunisation until the last visit<br>• Proportion of individuals carrying asexual parasites by microscopy at any density at Day 0 (before first administration), Week 4 (before second administration), 8, 26 (before booster dose), 30, 52 and 68<br>• Proportion of individuals carrying gametocytes by PCR at Day 0 (before 1st administration), Week 4 (before 2nd administration), 8, 26 (before booster dose), 30, 52 and 68<br>• The humoral response after natural boosting by measuring the titre of SE36 specific IgG by ELISA on samples obtained one week after each episode of clinical malaria occurring after the second immunisation until the last visit.<br>• Mapping of protective epitope(s) by ELISA against overlapping peptides derived from the SE36 protein at Week 8 and 30
**Statistical methods**	• For the primary outcome, the safety analysis population will include all subjects who received at least one injection (Intent-to-Treat population). Drop-outs data will be used until the date of discontinuation.<br>• Immunogenicity analysis will include all subjects who received at least one injection (Intent-To-Treat population) and will be performed at all-time points. A secondary analysis will include evaluated subjects who received the three doses of the allocated product according to protocol (Per Protocol population) and will be performed at all-time points.<br>• For the exploratory outcomes, statistical analyses will be exploratory, as the design is not powered to demonstrate statistically significant differences in outcomes.

	Interim Analysis<br>In order to assess the safety of the vaccine in young population without waiting until the end of the trial, an interim analysis will be prepared. The interim analysis will be on safety and IgG data generated until one month after the booster dose of cohort 1 and 2. The statistician will generate group results, while maintaining the blinding at individual participant level. However, in anticipation of AE requiring full description of the event, the trial will continue in a single blinded manner. The trial statistical analysis plan will reflect this interim analysis.<br><br>Final analysis<br>To ensure safety surveillance after the booster dose during the malaria transmission period, the trial will be extended for a further 4 months for each subject making a total follow up period of 16 months for each subject. A final statistical analysis will be carried out on all data generated during the trial until the end of the follow-up period.<br><br>Categorical variables will be summarised by vaccine groups as frequency, percentages and 95% confidence interval. In vaccine groups geometric means and 95% confidence intervals of the antibody titres will be determined. Continuous variables other than titres and concentrations will be summarised by vaccine groups as Mean, SEM, Median, Minimum, Maximum, inter-quartile range. The proportion of subjects that received three doses without experiencing Grade 3 adverse events will be estimated by Exact Binomial proportion (Proportion and 95% confidence interval).

2 Supplementary Tables

Supplementary Table S1. Immediate reactogenicity (first 60 minutes) after each vaccine dose.

	Cohort 1 (25-60 months)								
	Dose 1			Dose 2			Dose 3		
	BK IM	BK SC	Control	BK IM	BK SC	Control	BK IM	BK SC	Control
n	18	18	18	18	18	18	15	18	18
Abnormal pulse	4 (22%)* (6% to 48%)	5 (28%) (10% to 53%)	3 (17%) (4% to 41%)	5 (28%)* (10% to 53%)	4 (22%) (6% to 48%)	3 (17%) (4% to 41%)	1 (7%)* (0.2% to 32%)	0	1 (6%) (0.1% to 27%)
Abnormal temperature	0	0	0	0	0	0	0	0	0
Abnormal blood pressure	0	0	0	0	0	0	0	0	0
Pain/limitation of limb movement	0	1 (6%) (0.1% to 27%)	0	0	0	0	0	0	0
Local swelling	0	0	0	0	0	0	0	0	0
Local redness	0	0	0	0	0	0	0	0	0
Local induration	0	0	0	0	0	0	0	0	0
Loss of appetite	0	0	0	0	0	0	0	0	0
Irritability/fussiness	0	0	0	0	0	0	0	0	0
Drowsiness	0	0	0	0	0	0	0	0	0
Other AE	0	0	0	0	0	0	0	0	0

	Cohort 2 (12-24 months)								
	Dose 1			Dose 2			Dose 3		
	BK IM	BK SC	Control	BK IM	BK SC	Control	BK IM	BK SC	Control
n	18	18	18	18	18	18	18	17	18
Abnormal pulse	5 (28%) (10% to 53%)	1 (6%) (0.1% to 27%)	3 (17%) (4% to 41%)	1 (6%) (0.1% to 27%)	6 (33%) (13% to 59%)	3 (17%) (4% to 41%)	4 (22%) (6% to 48%)	3 (18%) (4% to 43%)	7 (39%) (17% to 64%)
Abnormal temperature	0	0	0	0	0	0	0	0	0
Abnormal blood pressure	0	0	0	0	0	0	0	0	0
Pain/limitation of limb movement	0	0	0	0	0	0	0	0	0
Local swelling	0	0	0	0	0	0	0	0	0
Local redness	0	0	0	0	0	0	0	0	0
Local induration	0	0	0	0	0	0	0	0	0
Loss of appetite	0	0	0	0	0	0	0	0	0
Irritability/fussiness	0	0	0	0	0	0	0	0	0
Drowsiness	0	0	0	0	0	0	0	0	0
Other AE	0	0	0	0	0	0	0	0	0

**no. of children experiencing an event (% of children along with the (95% CI)); BK IM, BK-SE36 via intramuscular route; BK SC, BK-SE36 via subcutaneous route*

Supplementary Table S2. Reactogenicity from Day 1-6 After Each Vaccine Dose.

	Dose 1		Dose 2		Dose 3	
	**BK-SE36 n = 72**	**Control* n = 36**	**BK-SE36 n = 72**	**Control n = 36**	**BK-SE36 n = 68**	**Control n = 36**
**Solicited local events**						
Swelling present	15 (21%)**	8 (22%)	21 (29%)	1 (3%)	12 (18%)	7 (19%)
Redness present	7 (10%)	2 (6%)	12 (17%)	2 (6%)	9 (13%)	1 (3%)
Induration present	48 (67%)	19 (53%)	43 (60%)	6 (17%)	36 (53%)	8 (22%)
Pain/limitation of limb movement	46 (64%)	24 (67%)	44 (61%)	8 (22%)	18 (26%)	5 (14%)
**Solicited system events**						
Fever measured	3 (4%)	4 (11%)	5 (7%)	1 (3%)	0	0
Fever reported	6 (8%)	2 (6%)	2 (3%)	1 (3%)	2 (3%)	0
Loss of appetite	2 (3%)	1 (3%)	2 (3%)	1 (3%)	1 (1.5%)	0
Irritability/fussiness	0	0	0	0	1 (1.5%)	0
Drowsiness	0	2 (6%)	4 (6%)	1 (3%)	0	0

**Control arm was vaccinated with Synflorix® at Dose 1 & 3 and physiological saline at Dose 2*

***no. of children experiencing an event (% of children)*

Supplementary Table S3. Summary of children that received three full doses of BK-SE36 and had Grade 3 and/or serious adverse events (SAE).

**Cohort 1**			
	BK-SE36 intramuscular	BK-SE36 subcutaneous	Control
Number of children receiving all three doses	15*	18	18
Grade 3/SAE events		2 (SAE: severe malaria)	1 (fever post Dose 1 and SAE: severe malaria)<br>1 (SAE: severe malaria)
Number (%) of children experiencing a Grade 3/SAE event	0 (0%)	2 (11%)	2 (11%)
Number (%) of children not experiencing a Grade 3/SAE event	15 (100%)<br>(97.5% CI, 78–100%)**	16 (89%)<br>(95% CI, 65–99%)	16 (89%)<br>(95% CI, 65–99%)

**Cohort 2**			
	BK-SE36 intramuscular	BK-SE36 subcutaneous	Control
Number of children receiving all three doses	18	17***	18
Grade 3/SAE events	1 (fever post Dose 2 and SAE: severe malaria)<br>2 (SAE: severe malaria)	1 (high transaminasemia)<br>2 (SAE: severe malaria)	1 (fever post Dose 1)<br>2 (SAE: severe malaria)
Number (%) of children experiencing a Grade 3/SAE event	3 (17%)	3 (18%)	3 (17%)
Number (%) of children not experiencing a Grade 3/SAE event	15 (83%)<br>(95% CI, 59–96%)	14 (82%)<br>(95% CI, 57–96%)	15 (83%)<br>(95% CI, 59–96%)

** 3 subjects received only 2 doses*

*** One-sided 97.5% confidence interval*

**** 1 subject received only 2 doses*

Supplementary Table S4. Fold-change (relative to Day 0) in total anti-SE36 IgG antibody titer.

Cohort 1	BK-SE36						Control (Synflorix®) Intramuscular	
	Intramuscular		Subcutaneous		Combined			
	n	Foldchange (95% CI)	n	Foldchange (95% CI)	n	Foldchange (95% CI)	n	Foldchange (95% CI)
Day 28*	18	1.9 (1.2, 3.2)	18	2.0 (1.1, 3.3)	36	2.0 (1.4, 2.8)	18	1.8 (1.0, 3.1)
Day 56	18	10.4 (5.2, 20.6)	18	7.3 (3.5, 15.1)	36	8.7 (5.4, 14.0)	18	1.7 (1.0, 3.1)
Day 182*	18	3.5 (2.2, 5.5)	18	2.9 (1.4, 5.8)	35	3.2 (2.1, 4.7)	18	1.7 (0.9, 3.4)
Day 210	17	16.5 (8.2, 33.0)	18	11.2 (5.1, 24.6)	35	13.5 (8.1, 22.4)	18	1.4 (0.7, 3.1)
Day 365	17	3.0 (1.3, 6.8)	18	2.5 (1.4, 4.6)	35	2.7 (1.7, 4.4)	17	1.0 (0.5, 2.0)
Day 477	17	4.1 (1.6, 10.0)	18	3.7 (1.5, 9.0)	35	3.9 (2.1, 7.0)	17	2.5 (1.1, 5.5)

*Subjects were vaccinated at Day 0, 28 and 182; n, number of subjects

p = 0.61 for comparison of BK-SE36 arms at D182 (prior to Dose 3) and *p* = 0.44 for comparison of BK-SE36 arms at D210 (4 weeks post Dose 3).

Cohort 2	BK-SE36						Control (Synflorix) Intramuscular	
	Intramuscular		Subcutaneous		Combined			
	n	Foldchange (95% CI)	n	Foldchange (95% CI)	n	Foldchange (95% CI)	n	Foldchange (95% CI)
Day 28*	18	3.9 (1.8, 8.6)	18	2.6 (1.3, 4.9)	36	3.2 (1.9, 5.2)	18	1.3 (0.7, 2.5)
Day 56	17	16.8 (6.5, 43.3)	16	10.6 (4.5, 24.8)	33	13.4 (7.3, 24.7)	18	0.8 (0.4, 1.5)
Day 182*	18	1.3 (0.5, 3.6)	17	1.1 (0.6, 2.0)	35	1.2 (0.7, 2.1)	18	0.4 (0.2, 0.7)
Day 210	18	37.6 (12.7, 111.1)	16	22.3 (8.7, 56.9)	34	29.4 (14.7, 58.7)	18	0.4 (0.2, 0.7)
Day 365	18	6.5 (2.3, 17.9)	16	3.6 (1.2, 11.1)	34	4.9 (2.4, 10.1)	18	1.1 (0.5, 2.2)
Day 477	18	5.9 (2.0, 17.1)	16	1.5 (0.6, 3.6)	34	3.1 (1.5, 6.3)	17	0.6 (0.3, 1.1)

* Subjects were vaccinated at Day 0, 28 and 182; n, number of subjects

p = 0.77 for comparison of BK-SE36 arms at D182 (prior to Dose 3) and *p* = 0.45 for comparison of BK-SE36 arms at D210 (4 weeks post Dose 3).

Supplementary Table S5. T-cell (IL-5, IL-13 and IFNɣ) cytokine responses per arm and per visit.

		Cohort 1				Cohort 2			
		BK-SE36 Combined		Control		BK-SE36 Combined		Control	
		n	GMC (95% CI)	n	GMC (95% CI)	n	GMC (95% CI)	n	GMC (95% CI)
IL-5	Day 0	3	9.1 (2.0, 40.9)	1	5.0	11	7.6 (3.1, 19.1)	2	6.1 (4.8, 7.7)
	Day 56	3	8.2 (3.4, 19.7)	0		9	4.9 (2.2, 10.9)	4	5.9 (0.6, 53.2)
	Day 210	1	2.3	1	3.0	6	7.5 (2.7, 21.2)	1	2.65
	Day 365	1	2.4	1	3.7	12	7.3 (4.4, 12.0)	3	6.5 (0.5, 81.6)
	Day 477	1	2.9	1	3.1	11	6.4 (3.9, 10.5)	8	4.3 (2.4, 7.6)
IL-13	Day 0	6	18.7 (3.0, 116.6)	4	50.1 (11.5, 218)	18	13.8 (8.3, 23.2)	12	18.8 (8.6, 41.1)
	Day 56	9	20.5 (5.2, 80.5)	4	18.4 (1.7, 195)	16	13.4 (6.7, 26.8)	10	11.7 (4.0, 34.8)
	Day 210	5	51.9 (7.5, 358.3)	5	41.2 (5.2, 329)	12	17.0 (8.4, 34.4)	6	5.5 (1.9, 16.5)
	Day 365	5	47.2 (14.6, 153.3)	6	52.1 (17.0, 159)	10	16.8 (6.9, 40.7)	9	13.1 (4.4, 39.3)
	Day 477	4	31.9 (3.2, 316.7)	2	28.7 (21.6, 38.0)	9	8.3 (4.1, 16.9)	4	5.4 (1.0, 31.0)
IFNɣ	Day 0	5	9.2 (4.1, 21.0)	1	4.0	7	18.0 (4.4, 73.7)	7	5.6 (2.3, 13.9)
	Day 56	4	14.6 (5.1, 41.9)	1	19.7	6	20.6 (3.0, 139.9)	3	29.9 (0.6, 1389)
	Day 210	3	9.9 (0.1, 1115)	1	17.4	8	12.1 (4.6, 31.7)	1	3.6
	Day 365	1	25.0	1	5.3	11	13.9 (7.3, 26.5)	3	44.2 (0.2, 7978)
	Day 477	1	6.2	0		4	13.3 (2.8, 63.5)	2	8.0 (6.4, 9.9)

**Day 56 and Day 210, 4 weeks after Dose 2 and 3, respectively*

GMC, geometric mean concentration (95% confidence interval); Combined, GMCs regardless of vaccination route

n, number of subjects

2.1 Supplementary Figures

Supplementary Figure 1. Sera from vaccinees reacted mostly to peptides corresponding to disordered regions of SE36. (A) Box-whisker plots of normalised ODs (at 492 nm) for the 15 peptides, Cohort 1 at D56 (4 weeks post Dose 2) and D210 (4 weeks post Dose 3). Green = control, blue = BK IM and red = BK SC. (B) Box-whisker plots of Cohort 2. (C) Structure prediction of SE36 and peptides used in epitope mapping. Blue arrow on top of sequence = β-strand; red coils = α-helix; grey highlights on sequence = predicted ordered regions; orange line below the sequence = octamer repeats; green line below the sequence = serine rich region; brown lines below the sequence = relatively conserved regions in SERA family. Numbered black arrows = position and sequence of designed peptides for epitope mapping.

(A) Cohort 1, 25- to 60-month-old

Day 56 (4 weeks post Dose 2)

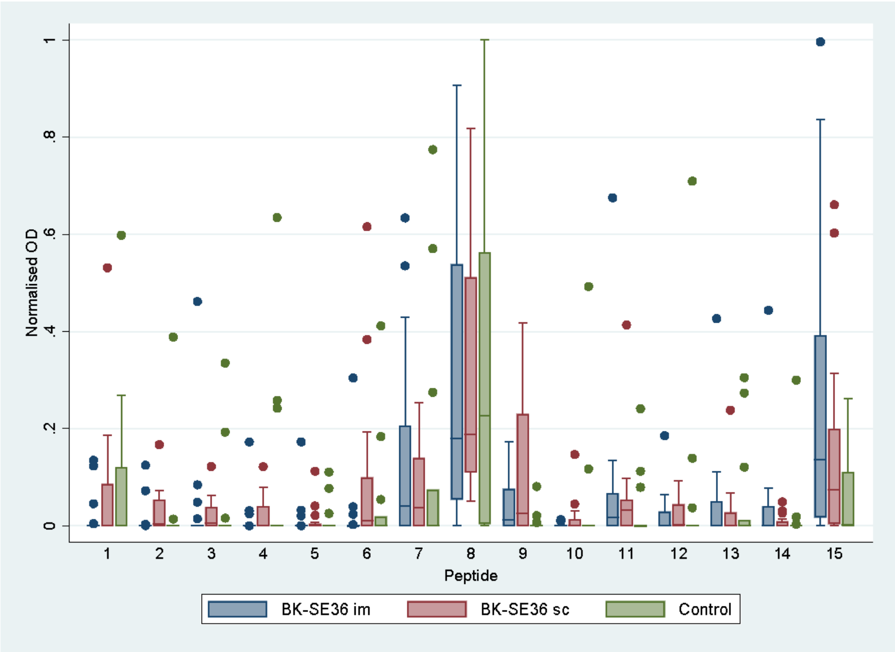

Day 210 (4 weeks post Dose 3)

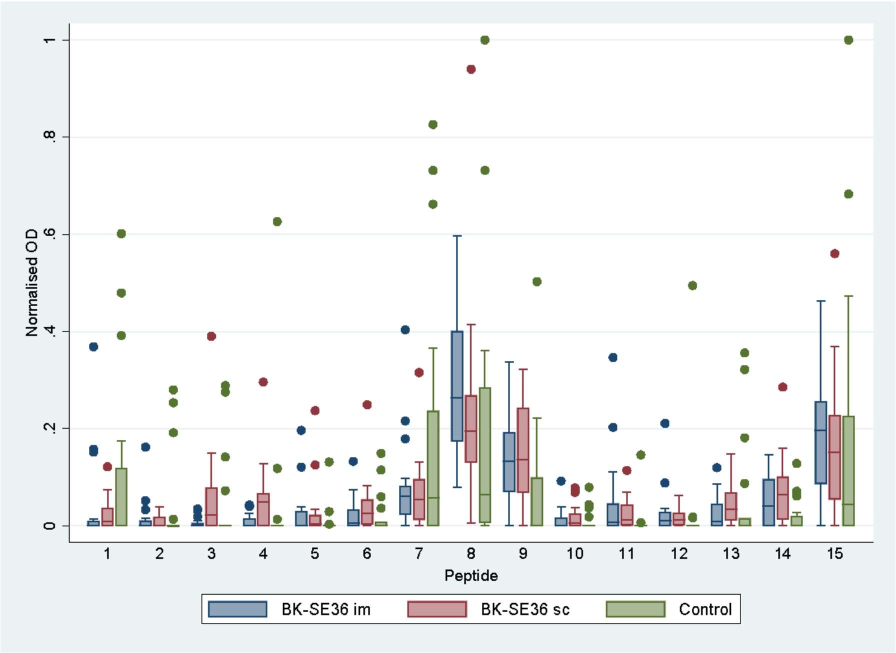

(B) Cohort 2, 12- to 24-month-old

Day 56 (4 weeks post Dose 2)

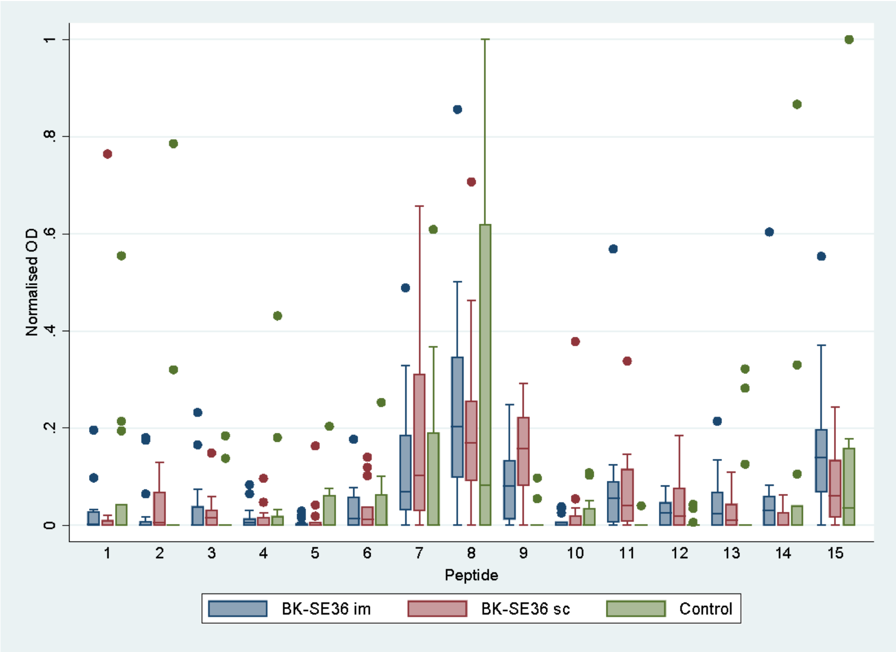

Day 210 (4 weeks post Dose 3)

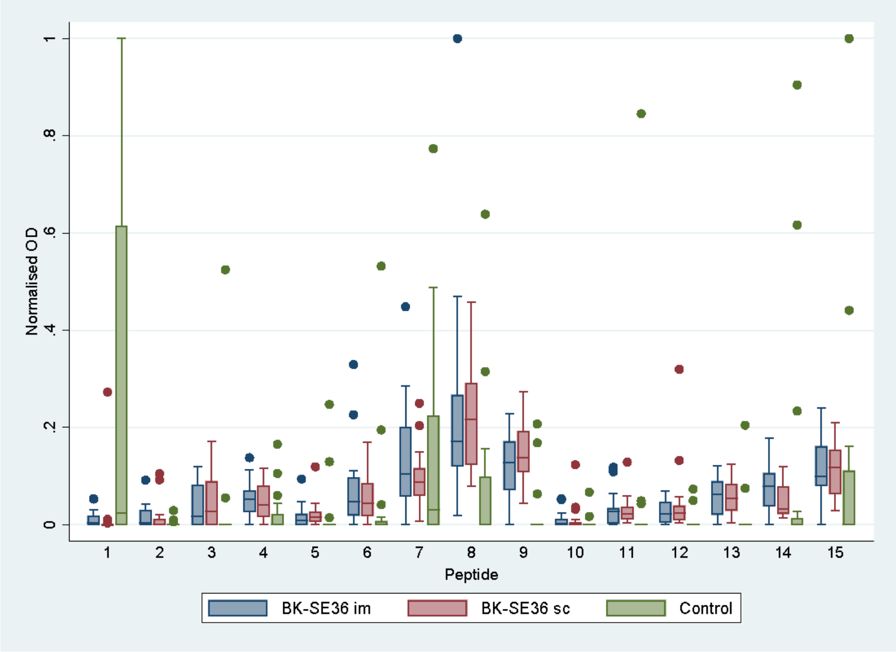

(C)

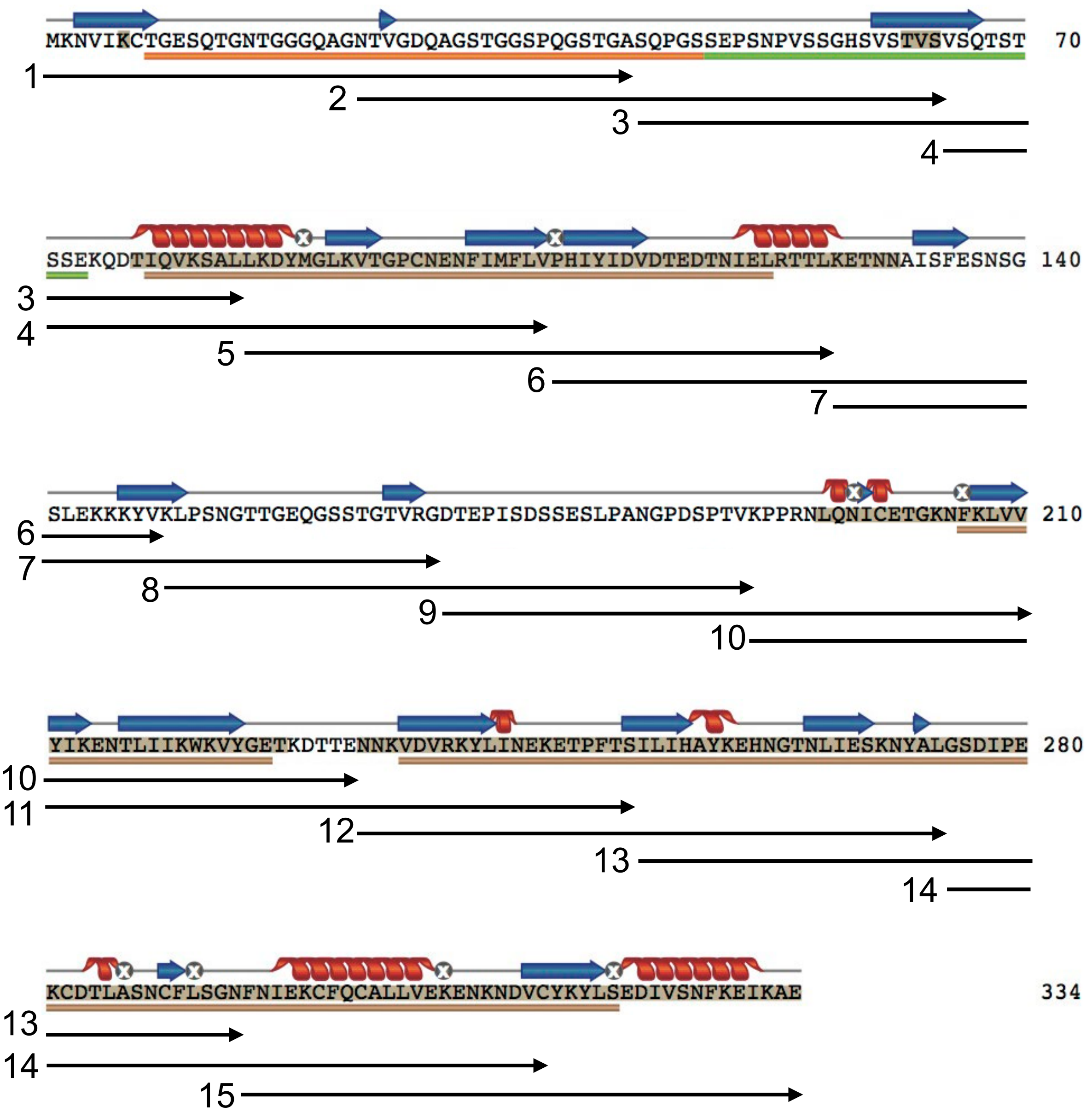

(SE36 structure prediction from *Yagi M, et al. PLoS One. 2014;9(6):e98460. doi:10.1371/journal.pone.0098460*)
